# Clubfoot from past to the present: A bibliometric analysis with global productivity and research trends

**DOI:** 10.1097/MD.0000000000032803

**Published:** 2023-02-10

**Authors:** Hakan Yolaçan, Serkan Güler, Ramadan Özmanevra

**Affiliations:** a Aksaray Training and Research Hospital, Orthopaedics and Traumatology, Aksaray, Turkey; b Cyprus International University, Orthopaedics and Traumatology, Nicosia, Cyprus.

**Keywords:** bibliometric analysis, clubfeet, clubfoot, *talipes equinovarus*

## Abstract

Clubfoot, a common congenital abnormality, affects the lower extremities; however, the literature search revealed no bibliometric research on this subject. Thus, we aimed to holistically analyze scientific articles and reveal global productivity and trend issues. This study statistically analyzed 1417 published articles on clubfoot (1980–2021) from the Web of Science database. Bibliometric network visualization maps were created to reveal trend topics, citation analysis, and cross-country collaborations. The analysis was conducted using Spearman correlation analysis. An exponential smoothing estimator was used to predict article productivity. The United States of America (433, 30.5%), the United Kingdom (166, 11.7%), and India (107, 7.5%) are the top 3 countries contributing to the literature. The *Journal of Pediatric Orthopedics* (220 articles), the *Journal of Pediatric Orthopedics-Part B* (147 articles), and *Clinical Orthopedics and Related Research* (69 articles) are the top 3 most productive journals. Dobbs MB (34 articles) is the most active author, and Shriners Hospital Children (44 articles) is the most active institution. Bibliometric analysis revealed that recently studied trend topics included Pirani score, Dimeglio score, Ponseti method, Ponseti casting, tenotomy, recurrence, neglected, tendon transfer, bracing, gait, risk factors, pedobarography, complex clubfoot, and polymorphism. The most studied subjects included Ponseti technique, treatment/casting, recurrent/relapsed clubfoot, Pirani score, pediatrics/children, foot deformities, surgery, ultrasound, Achilles tendon/tenotomy, gait analysis, casting, outcomes, neglected clubfoot, and tenotomy. Research leadership was determined in the western and European countries and Canada in studies and scientific collaborations on clubfoot; its impact was remarkable in India, China, and Turkey.

## 1. Introduction

Clubfoot, also known as *talipes equinovarus*, is a structural deformity of the foot and ankle with the hindfoot equinus (plantar flexion), varus of the heel (internal rotation), supination, and adduction of the forefoot (plantar cavus) developing early in pregnancy.^[1,2]^ The deformity is classified into 3 different types: idiopathic (unknown cause), neurogenic (caused by nervous system status), and syndromic (related to an underlying syndrome).^[3]^ The severity of the deformity can range from mild to manipulation-resistant extremely rigid foot.

The exact genetic mechanism of clubfoot has not yet been determined, but the underlying pathogenesis remains a matter of scientific debate. However, a multifactorial etiological model, including both environmental and genetic factors, is likely.^[2,4]^

The incidence of clubfoot, which is one of the common birth defects, varies by geographic region. A systematic review of data published over the past 55 years shows a birth prevalence of clubfoot ranging from 0.5 to 2.0 cases/1000 live births (China: 0.51, Africa: 1.11, India: 1.19, Southeast Asia: 1.21, America: 1.74, and Turkey: 2.03 per 1000 live births).^[2]^

Bibliometrics is the analysis of scientific outputs using various statistical approaches.^[5,6]^ Bibliometric analysis is a method that has become popular in recent years for researching and analyzing large volumes of scientific data. Bibliometric analyzes summarize the intellectual structure of a field by analyzing the structural relationships between different research components (e.g., authors, countries, institutions, topics). Therefore, well-done bibliometric studies provide scientists with a single point of view.^[7,8]^ It allows us to discover emerging trends and influential articles in a particular research topic or research area while providing insights into how that area or topic could move forward. Researchers can also provide new ideas for their new studies by examining past and current research trends revealed as analysis results of many articles.^[5–8]^

In parallel with the need to analyze the increasing number of articles in the literature, bibliometric research was conducted on many different subjects in the field of medicine.^[5–8]^ Clubfoot is one of the most common congenital abnormalities affecting the lower extremity, but the literature revealed no related bibliometric research. Thus, this study aimed to holistically analyze scientific articles published on clubfoot from 1980 to 2021 using statistical methods and bibliometric approaches, including citation analysis, and reveal global productivity and trend issues.

## 2. Methods

### 2.1. Research strategy

Web of Science Core Collection (WoS by Clarivate Analytics) database was used for the literature review. The search process was determined as 1980 to 2021. All studies with the words talipes equinovarus, clubfoot, club foot, clubfeet, club feet, club-foot, club-feet in the title were accessed (accessed August 15, 2022). The required scan codes are (TI [*talipes equinovarus*] or TI [clubfoot] or TI [club foot] or TI [clubfeet] or TI [club feet] and DOP [1980–2021]) for researchers to obtain similar documents (findings may vary slightly according to different access dates).

Ethical approval was waived for this study because no patients were enrolled and public databases were used.

### 2.2. Statistical analysis

The VOSviewer (Version 1.6.18, Leiden University’s Center for Science and Technology Studies, Netherlands) package program was used for bibliometric analyzes and network maps, including citation analysis.^[9]^ Statistical analyzes were performed with the Statistical Package for the Social Sciences version 22.0 (IBM Corporation, Chicago, IL) package program. The conformity of the data to the normal distribution was examined with the Kolmogorov-Smirnov test. The relationship between the world article productivity on clubfoot and some economic indicators of countries (gross domestic product [GDP] and GDP per capita) were analyzed with the Spearman correlation coefficient (data were obtained from the world bank).^[10]^ The website (https://app.datawrapper.de) was used for world map drawing. The Exponential Smoothing estimator, in which seasonal correction is considered, was used in the Microsoft Office Excel program to estimate the number of articles that can be published in the coming years according to the number of past articles. *P* values of < 0.05 was accepted for a statistically significant relationship.

## 3. Results

The literature review revealed a total of 1845 publications on clubfoot in the WoS database published from 1980 to 2021. The distribution of these publications are articles (1417, 76.8%), meeting abstracts (132, 7.1%), review articles (99, 5.4%), letters (80, 4.3%), proceedings papers (46, 2.4%), and the rest are in other publication types, including editorial materials, note, book chapters, book review, discussion, news item, biographical-item). Bibliometric analyzes were conducted with 1417 articles out of a total of 1845 publications in the article publication category. Of these articles, 94% (n = 1333) are in English and the rest are in other languages (German: 42, French: 17, Russian: 13, Spanish: 4, Turkish: 3, Czech: 2, Portuguese: 2, Hungarian: 1). Almost all of the articles were scanned in the science citation index-expanded (n = 1239, 87.4%) and emerging sources citation index (166, 11.7%) and the remaining few studies were social sciences citation index, conference proceedings citation index, and book citation index. It was indexed in Index–Science.

### 3.1. Distribution of publications on clubfoot from past to present

**Figure 1.**
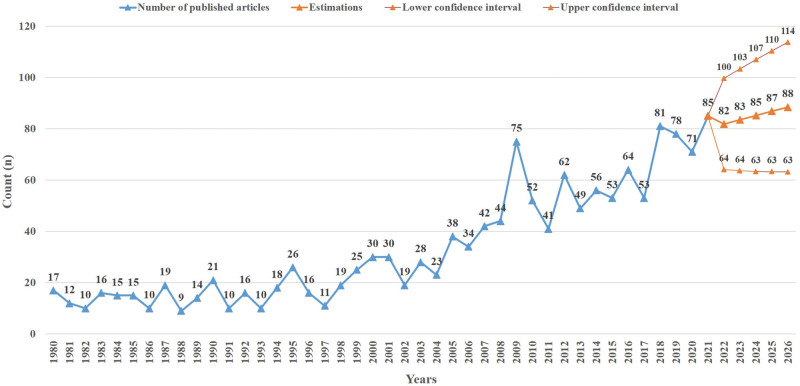
Bar graph showing the number of articles published in each year from 1980 to 2021, and the expected number of articles to be published over the following 5 years.

The distribution of the number of articles published on clubfoot by year is shown in Figure 1. The estimation values obtained by performing seasonal adjustment with the Exponential Smoothing estimation model, which is used to estimate the number of articles that can be published in 2022 and the next 5 years, are shown in Figure 1. According to the results of the model created, 82 (95% confidence interval: 64–100) estimated articles will be published in 2022 and 88 (95% confidence interval: 63–114) in 2026 (Fig. 1).

### 3.2. Countries that are prolific in clubfoot

**Figure 2.**
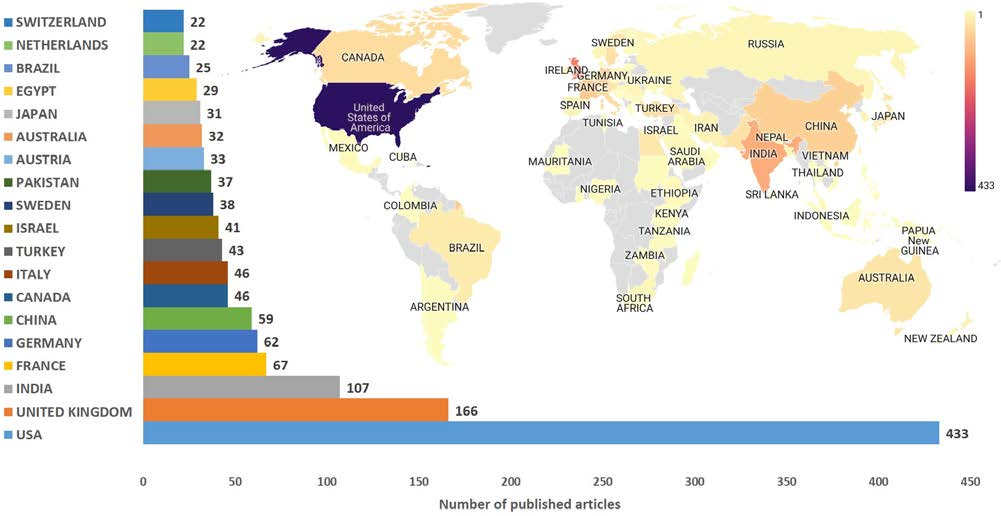
World article productivity map and bar chart for productive countries with the most articles on clubfoot.

**Figure 3.**
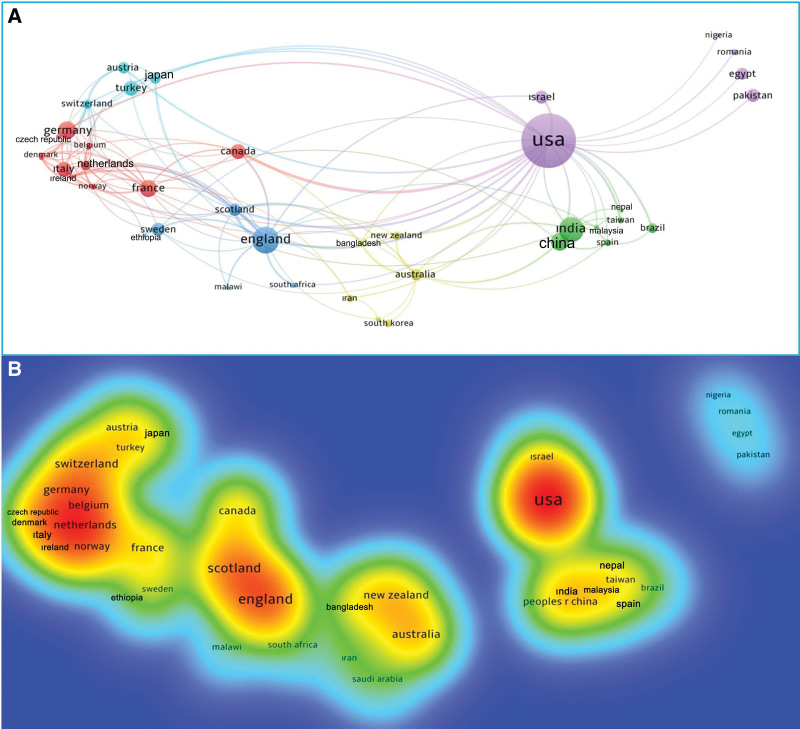
(A) Results of network visualization cluster analysis map showing international cooperation among countries on clubfoot. Different clusters are indicated by different colors. As the number of articles published by the countries increases, the area of the circles representing the countries increases. The lines show the countries with which they cooperate. (B) Density map showing the strength of international cooperation of countries on clubfoot. The strength of the international cooperation score increases from blue to red (blue-green-yellow-red).

The world productivity map showing the density of the number of articles by countries and the top 20 most productive countries are shown in Figure 2. Countries that contributed the most to the formation of the literature on clubfoot (with >20 articles published) included the United States of America (USA) (433, 30.5%), the United Kingdom (166, 11.7%), India (107, 7.5%), France (67, 4.7%), Germany (62, 4.3%), China (59, 4.1%), Canada (46, 3.2%), Italy (46, 3.2%), Turkey (43, 3%), Israel (41, 2.8%), Sweden (38, 2.6%), Pakistan (37, 2.6%), Austria (33, 2.3%), Australia (32, 2.2%), Japan (31, 2.1%), Egypt (29, 2%), Brazil (25, 1.7%), Netherlands (22, 1.5%), and Switzerland (22, 1.5%). Cluster analysis was performed among 40 countries that produced at least 5 articles from 89 countries that published articles on clubfoot and had international cooperation among their authors, as presented in Figure 3A. Six different clusters related to international cooperation were formed, including clusters 1 (red), 2 (green), 3 (blue), 4 (yellow), 5 (purple), and 6 (turquoise), according to the clustering analysis findings (Fig. 3A). Additionally, the total link strength scores showing the cooperation power of 40 countries in article productivity were calculated, and the international cooperation density map was created according to these scores, as presented in Figure 3B. The first countries with the highest score were the USA (82), England in the United Kingdom (47), Scotland in the United Kingdom (37), Australia (24), Switzerland (22), Canada (19), Netherlands (19), Ireland (19), France (18), New Zealand (18), and Germany (17).

### 3.3. Correlation analysis

A high level of statistically significant correlation was found between the number of articles produced by the countries on clubfoot and the GDP and GDP per capita values of the countries (*R* = 0.765, *P* < .001; *R* = 0.704, *P* < .001, respectively).

### 3.4. Active writers on clubfoot

The most prolific researchers who have published 15 or more articles on clubfoot are Dobbs MB (34 articles), Lehman WB (29 articles), Gurnett CA (23 articles), Morcuende JA (18 articles), Zionts LE (18 articles), Richards BS (17 articles), Ippolito E (16 articles), Karol LA (16 articles), Andriesse H (15 articles), Ebramzadeh E (15 articles), and Kuo KN (15).

### 3.5. Organizations active on clubfoot

The most prolific institutions that have published 20 or more articles on clubfoot are Shriners Hospital Children (44 articles), University of Iowa (42 articles), Washington University in St. Louis (35 articles), Udice French Research Universities (34 articles), Texas Scottish Rite Hospital for Children (33 articles), Assistance Publique Hopitaux Paris (30 articles), University of London (30 articles), University Paris Cite (29 articles), NYU Langone Medical Center (27 articles), University of California System (27 articles), Egyptian Knowledge Bank (25 articles), Hospital for Joint Disease NYU Langone Medical Center (25 articles), University of Aberdeen (21 articles), University of Texas System (21 articles), Harvard University (20 articles), New York University (20 articles), Tel Aviv University (20 articles), and University of California Los Angeles (20 articles).

### 3.6. Active magazines on clubfoot

In 313 different journals, 1417 articles on clubfoot were published. Among these journals, the most active 42 journals that have published >5 articles on clubfoot are shown in Table 1, as well as the total number of citations received by the journals and the average number of citations per article.

**Table 1:** The 42 most productive journals that have published more than 5 articles on clubfoot.

Journals	RC	C	AC	Journals	RC	C	AC
*Journal of Pediatric Orthopaedics*	220	5242	23.8	*Foot & Ankle*	10	128	12.8
*Journal of Pediatric Orthopaedics-Part B*	147	2273	15.5	*Bone & Joint Journal*	10	110	11
*Clinical Orthopaedics and Related Research*	69	1791	26	*Revue de Chirurgie Orthopedique et Reparatrice de L Appareil Moteur*	10	89	8.9
*Journal of Bone and Joint Surgery-British Volume*	62	2099	33.9	*Journal of The American Podiatric Medical Association*	10	40	4
*Journal of Childrens Orthopaedics*	56	577	10.3	*Travmatologiya I Ortopediya Rossii*	10	4	0.4
*Journal of Bone and Joint Surgery-American Volume*	45	3020	67.1	*Orthopedics*	9	39	4.3
*International Orthopaedics*	38	435	11.4	*Acta Orthopaedica Belgica*	8	42	5.3
*Journal of Foot & Ankle Surgery*	23	140	6.1	*American Journal of Medical Genetics*	7	259	37
*American Journal of Medical Genetics Part A*	17	303	17.8	*Plos One*	7	48	6.9
*Foot & Ankle International*	16	222	13.9	*Gait & Posture*	7	42	6
*Orthopade*	16	64	4	*Foot and Ankle Surgery*	7	36	5.1
*Acta Orthopaedica*	14	223	15.9	*Current Orthopaedic Practice*	7	13	1.9
*Indian Journal of Orthopaedics*	13	135	10.4	*Pediatric Radiology*	6	116	19.3
*Archives of Orthopaedic and Trauma Surgery*	12	189	15.8	*Journal of Ultrasound in Medicine*	6	73	12.2
*Prenatal Diagnosis*	12	136	11.3	*Journal of Orthopaedic Surgery and Research*	6	55	9.2
*Foot and Ankle Clinics*	12	84	7	*Tropical Doctor*	6	43	7.2
*Journal of Medical Genetics*	11	307	27.9	*Acta Ortopedica Brasileira*	6	23	3.8
*Acta Orthopaedica Scandinavica*	11	160	14.5	*Medicine*	6	23	3.8
*Bmc Musculoskeletal Disorders*	11	123	11.2	*Acta Orthopaedica et Traumatologica Turcica*	6	19	3.2
*Zeitschrift fur Orthopadie und Ihre Grenzgebiete*	11	49	4.5	*Journal of Clinical and Diagnostic Research*	6	12	2
*Pakistan Journal of Medical & Health Sciences*	11	1	0.1	*Journal of Evolution of Medical and Dental Sciences*	6	0	0

AC = average citation per document, C = number of citation, RC = record count.

### 3.7. Citation analysis on clubfoot

Table 2 shows the first 25 articles with the highest number of citations according to the total number of citations among the 1417 articles published on clubfoot. The average number of citations per year is given in the last column of Table 2.

**Table 2:** The top 25 most cited articles (more than 340 citations) published on clubfoot.

No	Article	Author	Journal	PY	TC	AC
1	Long-term results of treatment of congenital club foot	Laaveg SJ. and Ponseti IV.	*Journal of Bone and Joint Surgery-American Volume*	1980	429	9.98
2	Radical reduction in the rate of extensive corrective surgery for clubfoot using the Ponseti method	Morcuende JA. et al	*Pediatrics*	2004	392	20.63
3	Treatment of idiopathic clubfoot - a 30-year follow-up note	Cooper DM. and Dietz FR.	*Journal of Bone and Joint Surgery-American Volume*	1995	373	13.32
4	Classification of clubfoot	Dimeglio A. et al	*Journal of Pediatric Orthopaedics-Part B*	1995	359	12.82
5	Factors predictive of outcome after use of the Ponseti method for the treatment of idiopathic clubfeet	Dobbs MB. et al	*Journal of Bone and Joint Surgery-American Volume*	2004	285	15
6	Ponseti versus traditional methods of casting for idiopathic clubfoot	Herzenberg JE. et al	*Journal of Pediatric Orthopaedics*	2002	253	12.05
7	Long-term follow-up of patients with clubfeet treated with extensive soft-tissue release	Dobbs MB. et al	*Journal of Bone and Joint Surgery-American Volume*	2006	172	10.12
8	Long-term comparative results in patients with congenital club foot treated with two different protocols	Ippolito E. et al	*Journal of Bone and Joint Surgery-American Volume*	2003	160	8
9	Update on clubfoot: etiology and treatment	Dobbs MB. and Gurnett CA.	*Clinical Orthopaedics and Related Research*	2009	155	11.07
10	Early clubfoot recurrence after use of the Ponseti method in a New Zealand population	Haft GF. et al	*Journal of Bone and Joint Surgery-American Volume*	2007	145	9.06
11	The role of the Pirani scoring system in the management of club foot by the Ponseti method	Dyer PJ. and Davis N.	*Journal of Bone and Joint Surgery-British Volume*	2006	139	8.18
12	Genetic epidemiology study of idiopathic talipes equinovarus	Lochmiller C. et al	*American Journal of Medical Genetics*	1998	132	5.28
13	An independent assessment of two clubfoot-classification systems	Flynn JM. et al	*Journal of Pediatric Orthopaedics*	1998	131	5.24
14	Treatment of the complex idiopathic clubfoot	Ponseti IV. et al	*Clinical Orthopaedics and Related Research*	2006	126	7.41
15	A Comparison of two nonoperative methods of idiopathic clubfoot correction: the Ponseti method and the French functional (physiotherapy) method	Richards BS. et al	*Journal of Bone and Joint Surgery-American Volume*	2008	122	8.13
16	Deformity and disability from treated clubfoot	Aronson J. and Puskarich CL.	*Journal of Pediatric Orthopaedics*	1990	114	3.45
17	New concept of and approach to clubfoot treatment: section 2. Correction of the clubfoot	Mckay DW.	*Journal of Pediatric Orthopaedics*	1983	112	2.8
18	The Ilizarov distractor for the correction of relapsed or neglected clubfoot	Grill F. and Franke J.	*Journal of Bone and Joint Surgery-British Volume*	1987	110	3.06
19	A method for the early evaluation of the Ponseti (Iowa) technique for the treatment of idiopathic clubfoot	Lehman WB. et al	*Journal of Pediatric Orthopaedics-Part B*	2003	108	5.4
20	Congenital club foot in the human-fetus - histological study	Ippolito E. and Ponseti IV.	*Journal of Bone and Joint Surgery-American Volume*	1980	107	2.49
21	Treatment of idiopathic clubfoot using the Ponseti method: minimum 2-year follow-up	Abdelgawad AA. et al	*Journal of Pediatric Orthopaedics-Part B*	2007	106	6.63
22	Complete subtalar release in club feet. Part I. A preliminary-report	Simons GW.	*Journal of Bone and Joint Surgery-American Volume*	1985	102	2.68
23	Predicting the need for tenotorny in the Ponseti method for correction of clubfeet	Scher DM. et al	*Journal of Pediatric Orthopaedics*	2004	101	5.32
24	Magnetic resonance imaging study of the congenital clubfoot treated with the Ponseti method	Pirani S. et al	*Journal of Pediatric Orthopaedics*	2001	100	4.55
25	Correction of neglected idiopathic club foot by the Ponseti method	Lourenco AF. and Morcuende JA.	*Journal of Bone and Joint Surgery-British Volume*	2007	99	6.19

AC = average citation per year, PY = publication year, TC = total citation.

### 3.8. Co-citation analysis on clubfoot

All of the 1417 articles published on clubfoot had a total of 10,170 studies cited in the references section. Among these studies, the top 10 most co-citations (with >150 citations) were Cooper and Dietz (1995) (271 citations), Dobbs et al (2004) (219 citations), Dimeglio et al (1995) (279 citations), Herzenberg et al (2002) (199 citations), Laaveg and Ponseti (1980) (328 citations), Morcuende et al (2004) (290 citations), Ponseti (1996) (218 citations), Ponseti and Smoley (1963) (191 citations), Ponseti (1992) (212 citations), and Turco (1979) (152 citations).^[11–20]^

### 3.9. Trending topics on clubfoot

**Figure 4.**
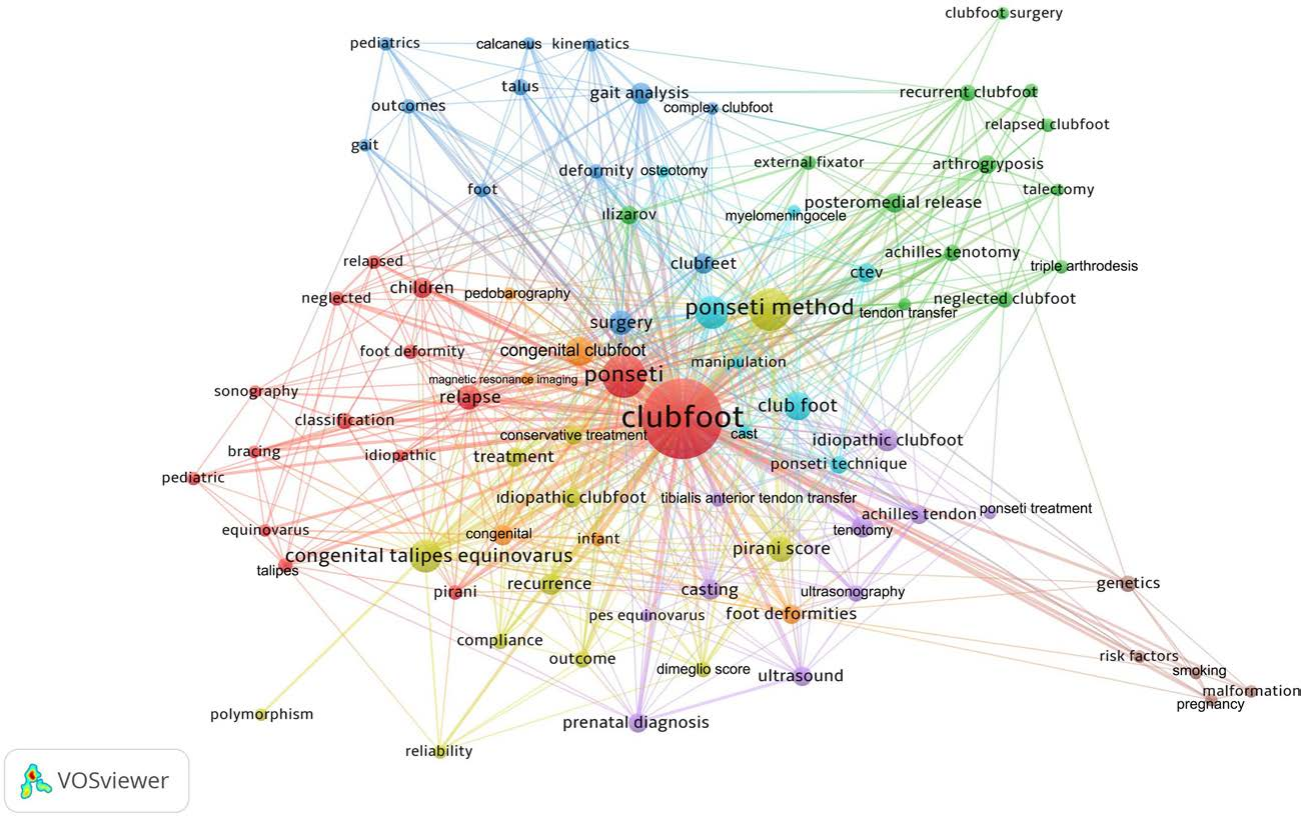
Network visualization cluster analysis map was created to show how clubfoot topics tend to cluster based on keyword analysis. Different clusters are indicated by different colors. Similar colored keywords are found in the same clusters. The number of keyword uses is indicated by the size of the circle.

**Figure 5.**
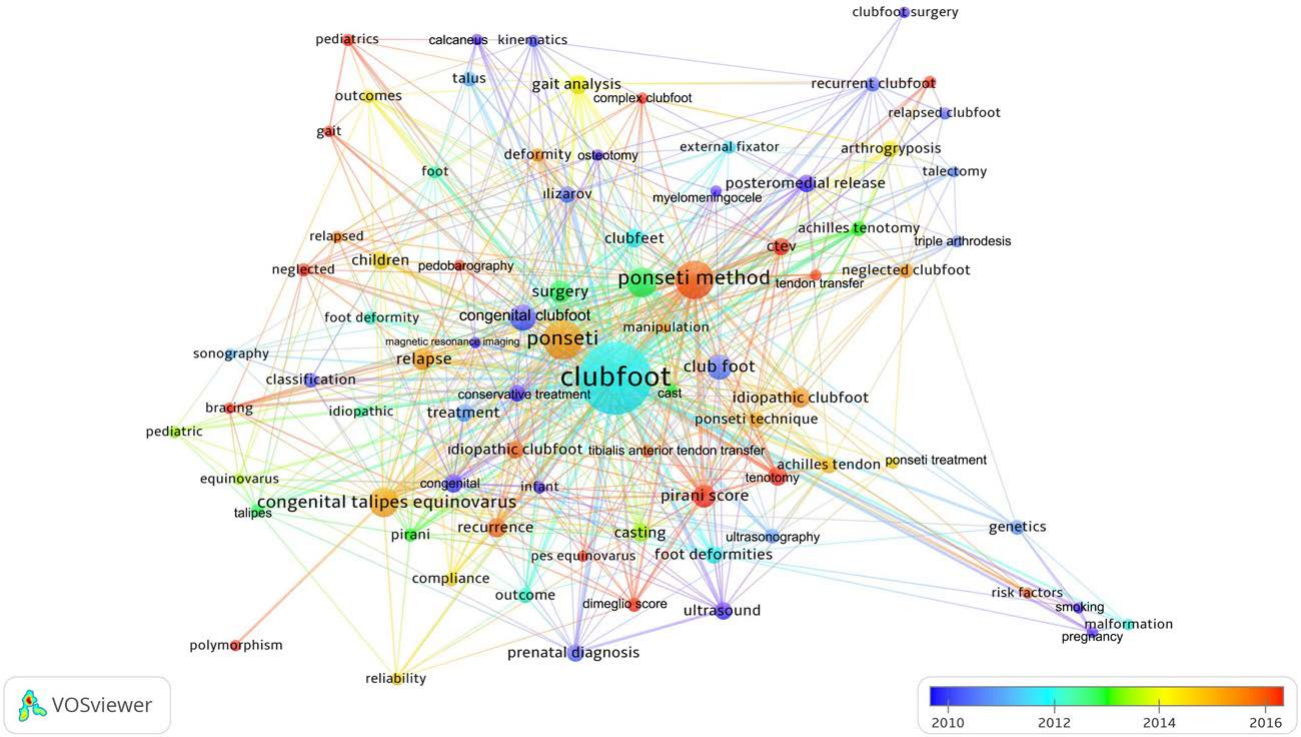
Past and current trends on clubfoot are displayed on a network visualization map based on keyword analysis. The indicator in the figure’s lower right corner changes from blue to red as the keyword becomes more current (blue-green-yellow-red). The number of keyword uses is indicated by the size of the circle.

**Figure 6.**
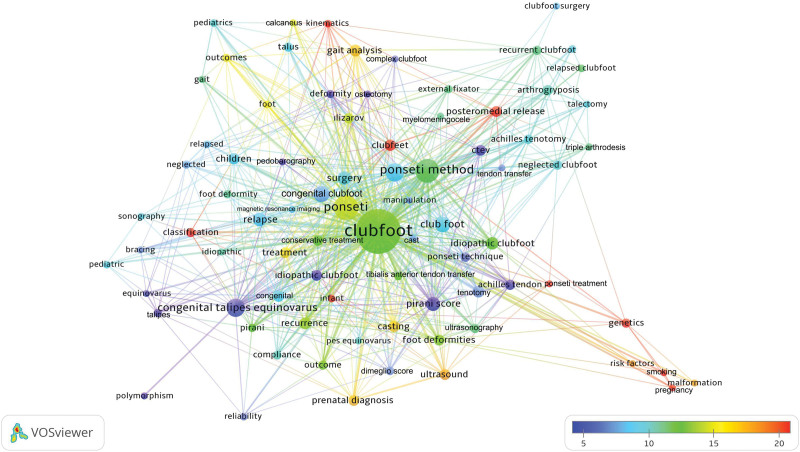
Most cited topics on clubfoot are displayed on a network visualization map based on keyword analysis. The indicator in the figure’s lower right corner changes from blue to red (blue-green-yellow-red) as the topic receives more citations. The size of the circle indicates the number of keyword uses.

All of the 1417 articles published on clubfoot used 1731 different keywords. Among these keywords, 81 different keywords used in >5 different articles are shown in Table 3. The cluster network visualization map showing the results of clustering analysis performed between these keywords is presented in Figure 4. A trend network visualization map made to reveal trend topics is presented in Figure 5 and a citation network visualization map made to identify the most cited topics is presented in Figure 6.

**Table 3:** The 97 most frequently used keywords in published articles on clubfoot.

Keywords	Number of uses	Keywords	Number of uses	Keywords	Number of uses
clubfoot	553	Ilizarov	15	relapsed	7
Ponseti	125	arthrogryposis	14	relapsed clubfoot	7
Ponseti method	115	Ponseti technique	13	reliability	7
congenital talipes equinovarus	62	achilles tenotomy	12	triple arthrodesis	7
talipes equinovarus	60	neglected clubfoot	12	bracing	6
congenital clubfoot	47	outcome	12	calcaneus	6
club foot	40	recurrent clubfoot	12	cast	6
Pirani score	32	talus	12	clubfoot surgery	6
relapse	30	ultrasonography	12	complex clubfoot	6
surgery	29	classification	11	equinovarus	6
idiopathic clubfoot	24	genetics	11	gait	6
recurrence	21	compliance	10	idiopathic	6
gait analysis	20	Dimeglio score	10	magnetic resonance imaging	6
tenotomy	20	deformity	9	malformation	6
casting	19	outcomes	9	manipulation	6
children	19	Pirani	9	myelomeningocele	6
clubfeet	19	external fixator	8	osteotomy	6
congenital	19	foot deformity	8	pedobarography	6
idiopathic clubfoot	17	neglected	8	pes equinovarus	6
prenatal diagnosis	17	talipes	8	polymorphism	6
ultrasound	17	tibialis anterior tendon transfer	8	Ponseti treatment	6
CTEV	16	foot	7	pregnancy	6
foot deformities	16	infant	7	risk factors	6
posteromedial release	16	kinematics	7	smoking	6
treatment	16	pediatric	7	sonography	6
achilles tendon	15	pediatrics	7	talectomy	6
conservative treatment	15	Ponseti casting	7	tendon transfer	6

## 4. Discussion

We identified the research leadership of Western countries, especially the USA, European countries, and Canada in research and scientific collaborations on clubfoot, but its impact was remarkable in developing countries, such as India, China, and Turkey.

The distribution of the articles published from 1980 to 2021 on clubfoot revealed 2 different publication trends observed as 1980 to 2006 and 2007 to 2021. During the 1980 to 2006 period, an average of 19 articles (min-max: 9–38) were published. The trend of increase in the number of articles started in 2007 and an average of 60 articles (min–max: 41–85) were published between 2007 and 2021. The estimated results for 2022 and the next 5 years revealed that the number of articles to be published on clubfoot will continue with an increase trend.

The article productivity of the world countries revealed that 13 of the most productive countries that contribute the most to the literature on clubfoot are developed countries, including the USA, United Kingdom, France, Germany, Canada, Italy, Israel, Sweden, Austria, Australia, Japan, Netherlands, and Switzerland, and Pakistan, India, China, Turkey, and Brazil, except Egypt, were developing countries. Among the developing countries, India, China, Turkey, and Brazil were countries with large economies. The high level of significant correlation between the number of articles published by countries on clubfoot and GDP and GDP per capita values suggests that the economic development level of countries is primarily effective in the productivity of global articles on clubfoot. Many bibliometric studies in the literature emphasized the positive effect of the development level of countries on academic productivity.^[5,7,8]^

The density map created according to the cooperation density between the countries determined that the countries with the highest cooperation were the USA, England in the United Kingdom, Scotland in the United Kingdom, Australia, Switzerland, Canada, Netherlands, Ireland, France, New Zealand, and Germany. The international co-authorship cooperation of the countries on clubfoot mentioned the effect of the geographical neighborhood in the studies (countries located in the same cluster and are geographically close include Iran and Saudi Arabia; Australia and New Zealand; England and Scotland; Austria and Switzerland; Germany, France, Belgium, Italy, Netherlands, Norway, Denmark, Poland, and Czech Republic; China, Nepal, and India).

The journals that contributed the most to the formation of the literature on clubfoot are *Journal of Pediatric Orthopedics, Journal of Pediatric Orthopedics-Part B, Clinical Orthopedics and Related Research, Journal of Bone and Joint Surgery-British Volume, Journal of Children’s Orthopedics*, and *Journal of Bone and Joint*. It was designated as *Surgery-American Volume, International Orthopedics*, and *Journal of Foot & Ankle Surgery*. This study recommends that researchers should first consider these journals to publish their studies on this subject. The average number of citations per article published by the journals revealed that the most influential journals are *Pediatrics* (the average number of citations the journal receives per article: 392), *Human Mutation* (73 citations), *Journal of Bone and Joint Surgery-American Volume* (67 citations), *American Volume. Journal of Human Genetics* (60 citations), *Techniques in Orthopedics* (44 citations), *Israel Medical Association Journal* (44 citations), *American Journal of Epidemiology* (40 citations), *American Journal of Medical Genetics* (37 citations), *Pediatric Annals* (36 citations), *Journal of Bone and Joint Surgery-British Volume* (34 citations), *Disability and Rehabilitation* (34 citations), and *Ultrasound in Obstetrics & Gynecology* (30 citations). This study recommends that researchers who want their work to see more impact after publication should first consider these journals.

The sorted articles according to the total number of received citations determined that the most cited study was the article titled “Long-term results of treatment of congenital club foot,” by Laaveg and Ponseti (1980) published in the Journal of Bone and Joint Surgery-American Volume (11th), followed by “Radical reduction in the rate of extensive corrective surgery for clubfoot using the Ponseti method,” by Morcuende et al (2004)^[16]^ published in *Pediatrics*, “Treatment of idiopathic clubfoot - a 30 year follow-up note” by Cooper and Dietz (1995) published in the *Journal of Bone and Joint Surgery-American Volume*,^[11]^ “Classification of clubfoot” by Dimeglio et al (1995)^[13]^ published in the *Journal of Pediatric Orthopedics-Part B*, and “Factors predictive of outcome after use of the Ponseti method for the treatment of idiopathic clubfeet” by Dobbs et al (2004),^[12]^ in order.

The evaluated articles according to the average number of received citations per year revealed that the most effective study is by Morcuende et al (2004),^[16]^ followed by Dobbs et al (2004),^[12]^ Cooper and Dietz (1995),^[11]^ Dimeglio et al (1995),^[13]^ and Herzenberg et al (2002)^[14]^ entitled “Ponseti versus traditional methods of casting for idiopathic clubfoot” published in the Journal of Pediatric Orthopedics, in order.

According to the number of co-citations made in the references of all analyzed articles, Cooper and Dietz (1995), Dobbs et al (2004), Dimeglio et al (1995), Herzenberg et al (2002), Laaveg and Ponseti (1980), Morcuende et al (2004), Ponseti (1996), Ponseti and Smoley (1963), Ponseti (1992), and Turco (1979) were determined as the most influential studies.^[11–20]^ This study suggests that researchers interested in this subject should first read these prominent publications.

The interpretation of the keyword analysis findings from the cluster analysis revealed that the keywords used in the clubfoot articles formed clusters in 8 different colors (red, green, blue, yellow, purple, turquoise, orange, and brown for clusters 1–8, respectively). The most studied topics from past to present (used as keywords in >20 articles) included Ponseti technique, treatment, or casting (266 uses), recurrent or relapsed clubfoot (77 uses), Pirani score (41 uses), pediatrics or children (33 uses), foot deformities (31 uses), surgery (29 uses), ultrasound (29 uses), Achilles tendon/tenotomy (27 uses), gait analysis (26 uses), casting (25 uses), outcomes (21 uses), neglected clubfoot (20 uses), tenotomy (20 uses). The most cited keywords were genetics, classification, posteromedial release, Ponseti treatment, kinematics, smoking, pregnancy, infant, gait analysis, risk factors, prenatal diagnosis, ultrasound, and malformation. The bibliometric analysis findings revealed that the trend topics studied in recent years include Pirani score, Dimeglio score, Ponseti method, Ponseti casting, tenotomy, recurrence, neglected, tendon transfer, bracing, gait, risk factors, pedobarography, complex clubfoot, and polymorphism.

Our literature review could not find a comprehensive bibliometric study on clubfoot. Only Malik and Noordin (2019) identified the top 100 most cited studies on clubfoot.^[21]^ Our study focused on all aspects of clubfoot. This study is the first bibliometric research on clubfoot; thus, it is expressed as an important advantage. The presence of bibliometric analyzes on international collaborations, global productivity, and trend research can be expressed as other advantages of our study in addition to our citation and co-citation analyzes. Our study limitation is the limited database, which only includes the WoS database. However, bibliometric analysis is not preferred because citation analyzes cannot be performed in the Pubmed database. Some journals with low impact levels are also indexed in the Scopus database.^[22,23]^ The most important reason why we prefer the WoS database is that it indexes the articles published in journals with a higher impact level (journals scanned in science citation index-expanded and emerging sources citation index indexes) compared to other databases. Additionally, WoS was widely preferred in other bibliometric studies in the literature.^[5–8]^

## 5. Conclusion

Scientific production on clubfoot has chronologically increased over the years. There are significant international collaborations globally, but clubfoot research is thought to be encouraged, especially in underdeveloped countries. These results may provide new ideas for future research.

## Author contributions

**Conceptualization:** Hakan Yolaçan, Serkan Güler, Ramadan Özmanevra.

**Data curation:** Hakan Yolaçan, Serkan Güler, Ramadan Özmanevra.

**Formal analysis:** Hakan Yolaçan, Serkan Güler, Ramadan Özmanevra.

**Funding acquisition:** Hakan Yolaçan, Serkan Güler, Ramadan Özmanevra.

**Investigation:** Hakan Yolaçan, Serkan Güler, Ramadan Özmanevra.

**Methodology:** Hakan Yolaçan, Serkan Güler, Ramadan Özmanevra.

**Project administration:** Hakan Yolaçan, Serkan Güler, Ramadan Özmanevra.

**Resources:** Hakan Yolaçan, Serkan Güler, Ramadan Özmanevra.

**Software:** Hakan Yolaçan, Serkan Güler, Ramadan Özmanevra.

**Supervision:** Hakan Yolaçan, Serkan Güler, Ramadan Özmanevra.

**Validation:** Hakan Yolaçan, Serkan Güler, Ramadan Özmanevra.

**Visualization:** Hakan Yolaçan, Serkan Güler, Ramadan Özmanevra.

**Writing – original draft:** Hakan Yolaçan, Serkan Güler, Ramadan Özmanevra.

**Writing – review & editing:** Hakan Yolaçan, Serkan Güler, Ramadan Özmanevra.
